# Supplementary material for: Qualitative Insights into Key Angelman Syndrome Motor Related Concepts Reported by Caregivers—A Thematic Analysis of Semi-Structured Interviews
Source: Children (Basel). 2023 Aug 28;10(9):1462. doi: 10.3390/children10091462 (PMC10529730; doi:10.3390/children10091462)
Supplement: Supplementary file 1 [file children-10-01462-s001.zip › children-2552028-supplementary.pdf]

Supplementary Tables and Figures

Supplementary Figure S1: Caregiver ranking of impaired lower limb motor function burden compared to other AS symptoms stratified by ambulation status of individual with AS

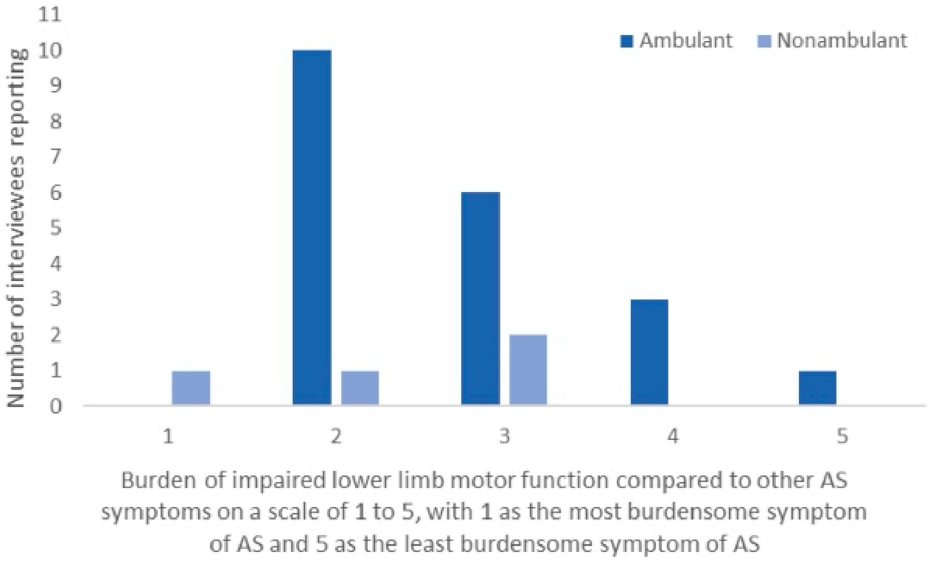

Supplementary Table S1: Most burdensome features of AS

Feature considered most burdensome	Quote to illustrate
**Gross motor**	*"Like, whether her motor function is the symptom that most affects us, the strongest one. I think it's the one that most affects us."*<br>- *Mother of a 4-year old female, Colombia*
**Cognitive development**	*"It's not so serious. Not as serious as his reasoning. Because if his reasoning was better, he could coordinate the rest, because he can already move on his own."*<br>- *Mother of a 6-year-old male, Colombia*
**Communication difficulties**	*"Any form of communication, but speech would be simple thing like yes and no, or hungry or bathroom. Very simple things would be an amazing improvement in his life and in our life."*<br>- *Father of a 5-year-old male, USA*
**Fine motor**	*"That [improvement of motor function] would not be the gross motor, but the fine motor skills. That's the eating part"*<br>- *Mother of a 4-year-old female, Belgium*
**Behavioural issues**	*"I don't know, because [she] is extremely aggressive, and she's nonverbal…. If you were going to rank them, those are ahead of the motor, but I would put it in the top five for sure."*<br>- *Father of an 11-year-old female, USA*

Supplementary Table S2: Caregiver-reported difficulties relating to AS medical management

Difficulty relating to medical management	Quote to illustrate
**Difficulty accessing medical care due to location**	*" Access to providers impacts the ability to get an appointment and the location of where you have to travel to get an appointment."*<br>- Clinician discussing an 8-year-old male, USA
**Difficulty accessing medical care due to timeframe**	*" The biggest thing is that there's physical therapy you can go to. Only if there's something that comes up then you can get a short-term order for it."*<br>- Mother of a 22-year-old female, USA
**Difficulty accessing medical care due to lack of specialists**	*" There are not many professionals specialised in this condition in kinesiotherapy. That's very clear. There are some paediatric kinesiologists. It's not easy to find professionals that understand kinesiotherapy for a child."*<br>- Mother of a 2.5-year-old male, France
**Lack of information on the condition**	*"Yes, quite a lot. There is a significant lack of information about this condition. It's a very unknown disease and the professionals don't know it. They treat it as if it were a different kind of disease or condition, but the results aren't the same."*<br>- Mother of a 6-year-old male, Colombia
**Difficulty in patients engaging in care**	*"The biggest issue was his ability to receive treatment or care. It's enough for him to be tired or not have a good day, for him to be hungry or something else, and that treatment is no good at all. You can try to do it with him and do the exercises, but he won't take it."*<br>- Mother of a 5-year-old male, Belgium

Supplementary Table S3: Quotes illustrating relevant variables to measure in AS

Variable	Quote to illustrate
**Walking perimeter**	*"...the perimeter seems important because it's even a little bit in the day. If she's not able to do that without stopping that could mean if we're at the hospital and she's seeing a doctor and have to walk into the bathroom and it's far away, that could be a challenge."*<br>- Aunt of a 20-year-old female, USA
**Stride length**	*"Length seems important to me because her steps are short and they're not normal. I wonder if she's really walking efficiently. She takes these little hard short steps and so it makes me feel she has to work harder to get where she needs to go."*<br>- Aunt of a 20-year-old female, USA
**Number of strides per day/Distance walked**	*"I don't know if the stride length is going to be how important that is. I would say it's the number of steps or the distance traveled that's going to be important than the length of the stride."*<br>- Mother of a 35-year-old male, USA
**Falls**	*"It seems huge. Because when she falls, she really hurts herself. She has the potential to really hurt herself depending on where she falls, because she tenses up and falls down hard. As I gave you the example, she falls on somebody and then that person gets hurt."*<br>- Aunt of a 20-year-old female, USA
**Stair climbing**	*"It's important because definitely she cannot navigate the stairs without assistance. If she was able to go up and down stairs by herself, that would be a major change."*<br>- Aunt of a 20-year-old female, USA<br><br>"*It would be helpful for going downstairs as he struggles more with going down than going up*."<br>- Mother of a 6-year-old male, USA
**Stride velocity**	*"Well, I mean the speed is a factor because it's very slow, too. I'm standing there helping her do it and that takes a lot of time. Change in speed would also be important."*<br>- Aunt of a 20-year-old female, USA

Supplementary Table S4: Other potential lower-limb motor function variables suggested by caregivers

[illegible]
Ataxia: Balance, Immobility standing without support, Gait deformation, Left/Right symmetry, Foot trajectory, Gait narrowing<br>Direction & speed variation
Differentiate data with/without assistance (number of falls, climb/descend speed), Assess walking independence
Fatigue
Time to assess next walking zone
Knee flexion & positioning
Surroundings impact the behavior and assistance (school, home, ice, grass, playground…)
Pre-gait development (pulling to stand, positionality, coordination)
Other: Arterial pressure, Feet pain, Predict epilepsy, Falls notifications, Ability to perform daily tasks (eating, showering…)

Supplementary Table S5: Positives of a wearable measure of motor function in AS and potential issues, according to caregivers

Concepts arisen	Quote
**Positives**	
**Current lack of objective measures**	*Interviewer: "So what you're saying right now is that there is no objective way of judging what the best options are for physical therapies and for drugs? Is that what you're saying?"*<br>*Interviewee: "Correct."*<br>- Sister of a 26-year-old male, USA
**Real-time data**	*"It particularly makes sense that it's on a sensor that is tangible and for which there is really reliable data. It's not just a subjective assessment. It's something tangible, rational, in fact, with data. It's reliable. That's very important."*<br>- Mother of a 6-year-old female, France
**Wearability**	*"Our first concern was that we thought it might be a burden or an additional handicap, but that wasn't the case at all. We spoke about it initially and we thought about how it was going to work. We said that they were going to take it off, that it wasn't going to stay on even five minutes. But that wasn't true at all. I think my child has got completely used to it. It was on his feet, but that didn't bother him."*<br>- Mother of a 5-year-old male, Belgium
**Potential issues**	
**Tolerance of wearable**	*"The real question would be to know whether she accepts it, whether she spends her time trying to take it off, either using her hands or moving her foot very firmly so that it falls off. It's important that it doesn't become something that bothers her. "*<br>- Mother of a 6-year-old female with a non-deletion, France<br>*"He won't keep anything on. We've tried trackers with some of the other research studies we've been involved with. The kids notoriously tear those things off."*<br>- Mother of a 35-year-old male, USA
**Limited movement**	*"The other measures are informative nonetheless, but you will get very limited data because he does so little."*<br>- Sister of a 26-year-old male, USA

**How to differentiate assisted vs not assisted**	"*I don't know how to do it, but what's important for me is to separate everything that you've said, when she's on her own from when she's with someone holding her.*"<br>- Mother of a 6-year-old female, France<br><br>"*Unless it's done with independence and that would have to be some home monitor or adjust. If we ever had to bring her upstairs and something was attached to her ankle or something had to be noted, like, hey, you know what? We pushed her up the stairs and she did it, and your device is going to recognize that, but you're going to have to know mom and dad helped or somebody helped, because she sure as heck ain't going to do it on her own.*"<br>- Father of an 11-year-old female, USA
**Side effects of medications**	"*I think it really depends if the child is having seizures and if the child is taking any medication. A lot of that medication too has effects on their muscles. They have a lot of negative side effects.*"<br>- Mother of a 5-year-old female, USA
**Effect of age**	"*It depends on how old they are because you can't measure that for a teenager because he's in his room.*"<br>- Mother of an 8-year-old male, Chile
**Difference in going up vs down stairs**	"*Going down is more difficult than going up. It would need to have that.*"<br>- Mother of a 6-year-old male, Colombia
**Impact of other symptoms**	"*Yes, but at the cognitive level, there is no planning. So the motor thing on its own means nothing. How can we separate just motor function?*"
	- Mother of a 5-year-old female, Chile
**Forgetting to put watch on**	"*Another thing that would also be good perhaps is to get reminders because, obviously, it happened that one day we realised that we'd forgotten to put it on in the morning.*"<br>- Mother of a 5-year-old male, Belgium
**Quality vs quantity of strides**	"*You can make a lot of strides, but poor strides. It's more about the quality of the strides than the number.*"<br>- Father of a 10-year-old male, UK
**Impact of tiredness (general exhaustion rather than specifically muscle fatigue)**	"*Yes, sometimes. I don't know if you can measure this using a device, but sometimes we don't know whether he doesn't want to walk because he's tired or because he doesn't want to.*" *Interviewer: "OK. So, having a measure of his level of tiredness." Mother: "That's it. That could be interesting to know whether it's because he's tired, whether it's a real physical incapacity or because he doesn't want to*"<br>- Mother of a 6-year-old male, Colombia
**Impact of environment**	"*It's like they walk quite straight. I'm thinking about what other things they do. What I notice is also a visual thing. When they're walking and they see that the colour or the texture of the floor changes. That's important to. They get to that point and they suddenly get up or do this, to see whether the relief of the floor changes.*"<br>- Mother of a 12-year-old male, USA
**Impact of climate or temperature**	"*Are your kids going to be outside? If you're in Texas, it's 110 degrees outside so you're going to have to keep them inside. It's a catch-22.*"<br>- Mother of a 5-year-old female, USA
